# Emerging Artificial Intelligence–Empowered mHealth: Scoping Review

**DOI:** 10.2196/35053

**Published:** 2022-06-09

**Authors:** Paras Bhatt, Jia Liu, Yanmin Gong, Jing Wang, Yuanxiong Guo

**Affiliations:** 1 Department of Electrical & Computer Engineering The University of Texas at San Antonio San Antonio, TX United States; 2 The University of Texas Health Science Center at San Antonio San Antonio, TX United States; 3 Florida State University Tallahassee, FL United States

**Keywords:** mobile health units, telemedicine, machine learning, artificial intelligence, review literature as topic

## Abstract

**Background:**

Artificial intelligence (AI) has revolutionized health care delivery in recent years. There is an increase in research for advanced AI techniques, such as deep learning, to build predictive models for the early detection of diseases. Such predictive models leverage mobile health (mHealth) data from wearable sensors and smartphones to discover novel ways for detecting and managing chronic diseases and mental health conditions.

**Objective:**

Currently, little is known about the use of AI-powered mHealth (AIM) settings. Therefore, this scoping review aims to map current research on the emerging use of AIM for managing diseases and promoting health. Our objective is to synthesize research in AIM models that have increasingly been used for health care delivery in the last 2 years.

**Methods:**

Using Arksey and O’Malley’s 5-point framework for conducting scoping reviews, we reviewed AIM literature from the past 2 years in the fields of biomedical technology, AI, and information systems. We searched 3 databases, PubsOnline at *INFORMS*, e-journal archive at *MIS Quarterly*, and Association for Computing Machinery (ACM) Digital Library using keywords such as “mobile healthcare,” “wearable medical sensors,” “smartphones”, and “AI.” We included AIM articles and excluded technical articles focused only on AI models. We also used the PRISMA (Preferred Reporting Items for Systematic Reviews and Meta-Analyses) technique for identifying articles that represent a comprehensive view of current research in the AIM domain.

**Results:**

We screened 108 articles focusing on developing AIM models for ensuring better health care delivery, detecting diseases early, and diagnosing chronic health conditions, and 37 articles were eligible for inclusion, with 31 of the 37 articles being published last year (76%). Of the included articles, 9 studied AI models to detect serious mental health issues, such as depression and suicidal tendencies, and chronic health conditions, such as sleep apnea and diabetes. Several articles discussed the application of AIM models for remote patient monitoring and disease management. The considered primary health concerns belonged to 3 categories: mental health, physical health, and health promotion and wellness. Moreover, 14 of the 37 articles used AIM applications to research physical health, representing 38% of the total studies. Finally, 28 out of the 37 (76%) studies used proprietary data sets rather than public data sets. We found a lack of research in addressing chronic mental health issues and a lack of publicly available data sets for AIM research.

**Conclusions:**

The application of AIM models for disease detection and management is a growing research domain. These models provide accurate predictions for enabling preventive care on a broader scale in the health care domain. Given the ever-increasing need for remote disease management during the pandemic, recent AI techniques, such as federated learning and explainable AI, can act as a catalyst for increasing the adoption of AIM and enabling secure data sharing across the health care industry.

## Introduction

Initially, information technology systems were mainly used to record patient data [1], but the rapid development in technology over the years has paved the way for data analytics and machine learning (ML) to be applied in the health care domain [2]. Advanced artificial intelligence (AI) techniques combined with the rapid integration of medical internet of things (IoT) devices [3] has led to an increase in research on digital health care and preventive medicine [4]. Such research focuses on mobile health (mHealth) technologies that are used to monitor serious ailments, like asthma, diabetes, and sleep apnea, and to ensure patient well-being and safety [5]. mHealth is a critical sector of the health care information technology industry that has grown rapidly in recent years [6]. This growth has been fueled by the rise in wearable technologies [7], mobile sensors [8], and the exponential increase in the number of IoT devices in general [9]. Such devices are increasingly used in hospitals and medical institutions [10] for constant patient monitoring [11] and intensive care unit capacity monitoring. Coinciding with the increase in the use of IoT devices, the wearing of health devices outside hospital premises for remote in-home care has also increased [12]. This has led to both a greater level of research [13] and higher investment in mHealth [14]. Researchers have stressed mHealth’s importance in challenging times such as the current pandemic to enable the provision of remote health care facilities [15]. Recent research indicates that there has been a significant increase in mHealth usage since COVID-19 [16]. AI has helped scholars to research new avenues of clinical care that are focused on ensuring the maintenance of social distancing and better hygiene and have developed remote mHealth capabilities that can enable patient care during and after COVID-19 [17,18].

With the increase in mHealth research, there have been significant improvements in the level of AI available to researchers as well. These improvements offer more accurate insights and results than does traditional ML while simultaneously preserving patient privacy and ensuring a high data security standard. Deep learning (DL) and federated learning (FL) [19,20] are some examples of these newer techniques that ensure data security and privacy. Consequently, researchers have used AI techniques to study novel scenarios and tasks within the health care IT domain, from using it to classify and predict disease occurrence [21], to detecting the presence of chronic illnesses [22], and even assisting doctors in making decisions about preventive health care programs [23]. AI has been successfully integrated with the health care sector, and many systematic literature surveys outline its importance to this domain [24-26].

Recently, research related to AI techniques in the mHealth sector has increased considerably [27]. This can be attributed to the rapid evolution and acceptance of telehealth during the COVID-19 pandemic [28]. As a result of several changes in telehealth policies (telehealth integration into hospital portals, expanding insurance coverage for telehealth services, and increasing patient choices for telehealth services) [29,30], telehealth has emerged as a viable alternative to providing care to noncritical patients [31], thus enabling hospitals and medical institutions to direct their resources to serving critical patients. The adoption of mHealth devices has also increased during this period [16], providing both localized and personalized patient information [32] and resulting in the generation of a large amount of data which is particularly well-suited to train AI models. ML algorithms running locally on smart and wearable devices have led to novel insights. For instance, researchers use AI to study neurogenerative disorders like Parkinson [33] and Alzheimer [34] disease, which exhibit latent temporal symptoms that are difficult to characterize without mHealth sensors. This symbiosis of AI and mHealth technologies is crucial for the development of remote health care infrastructure that can better inform physicians and benefit millions of patients.

Using mHealth sensors, researchers have documented disease progression [35], depicting how an illness spreads or manifests over time in a patient. These insights can be significant in the early diagnosis and treatment of chronic diseases and management of symptoms hitherto undetectable by traditional patient monitoring within hospitals and assisted living facilities. This confluence of AI and mHealth has given rise to a new domain of research that studies the combination of these 2 research streams. It is known as AI-powered mHealth (AIM) [36]. Using AI techniques in the application of mHealth scenarios can have numerous benefits, such as the automatic detection of chronic disease occurrence [21], real-time prediction and intervention for suicide prevention [37], facilitating emergency response [38], enabling patient rehabilitation [39], providing noninvasive care [40,41], and preventing medical errors. Preventable medical error is a significant cause of death in the USA. Clinical decision-making technology can significantly reduce it by using real-time data from wearable health sensors [42,43].

AIM devices can power ubiquitous health care solutions through remote patient monitoring [44], which is essential for providing health services in remote and medically underserved areas, where patients are not connected with modern health care systems. AIM can also enable at-risk minority populations who do not have access to health care facilities receive quality health care with ease [45,46]. With the development of newer AI techniques, such as DL, reinforcement learning, and few-shot learning, the domain of AIM will only grow in the future [47,48]. Furthermore, mHealth has implications for remote patient-monitoring and telehealth research and practice, which is becoming a reality much faster than the medical industry expected because of the COVID-19 pandemic [49].

Prior research focusing on the application of AI in the health care domain noted that certain implementation factors exist that prevent large scale automation of the health care sector [50]. However, with the advancement in AI techniques and the advent of DL, there has been a significant rise in both AIM research and practice. Previous surveys of mHealth have focused on only niche conditions, such as musculoskeletal medicine [51], or have attempted to study perceptions of AI in the mHealth domain and health care settings in general [52,53].

Over the last couple of years, there have been significant advances in both the usage of AI and mHealth. In this regard, several recent studies share an overlapping context (AI + mHealth) [54-57] in seeking to explain and implement the clinical use of AI in mHealth settings. A current review of such research is lacking, which presents a gap in the AI mHealth literature. Therefore, it is necessary to survey the current state of the art in AIM research (eg, current work, current solutions, and future opportunities) both in the mHealth industry and the field of AI. A scoping review of this research is much needed, as it addresses the gap in literature related to an in-depth analysis of AI capabilities currently being used in the mHealth settings. Our aim in this paper is to further expand the research scope of this critical health care domain and explore the opportunities for future development. To the best of our knowledge, this is the first attempt to survey research on AIM analytics. Our objective is also to map current research on the growing use of AIM for remote patient-monitoring and examine how researchers use patient data for building AI models for disease management.

## Methods

Scoping reviews are used to examine the extent, range, and nature of research activity in a particular domain. In this context, we used Arksey and O’Malley’s [58] 5-step scoping review framework to guide our search strategy for reviewing current peer-reviewed AIM research.

### Step 1: Identifying Research Questions

We started by identifying our research questions (RQs) and aimed to survey the literature on the current use of AIM to identify and manage different health conditions. We also investigated the use of data collected from wearable sensors and mobile devices for building AIM models.

### Step 2: Identifying Relevant Studies

After specifying our RQs, we identified relevant studies to be screened in this review. This involved searching electronic databases including PubsOnline at *INFORMS* and the e-journal archive at *MIS Quarterly* for information systems (IS) articles. We used the Association for Computing Machinery (ACM) Digital Library, which catalogs research from top conferences and journals in the AI domain, for AI articles. We also used a search query in Google Scholar with a 2-year filter (since 2019) for including recent articles on specific advancements in the field of AIM related to the use of FL and explainable AI (XAI). The state of the art in AI until 2019 had been covered by previous researchers in surveys and reviews on AI in the health care sector [50,51,53]; therefore, we decided to focus on articles from 2019 and beyond. Moreover, since DL has only been growing in the health care domain during the last couple of years and newer AIM techniques such as FL have recently emerged as privacy-preserving mechanisms, we decided to limit the search to articles published from 2019 to the present.

The articles screened for this review were published in the 3 major domains of biomedical technology, AI, and IS. In this regard, the journals and articles searched were from top venues in these domains. We searched the *Journal of Biomedical Informatics*, *Journal of Medical Internet Research*, and *Nature Medicine* for biomedical technology articles. For AI, we focused our efforts on recent top conferences including the Conference on Neural Information Processing Systems (NeurIPS) and the Association for the Advancement of Artificial Intelligence (AAAI). Within these conferences, we looked at ML for health, ML for mobile health, ML for public health, and web search and data mining. As for IS, we searched articles in the top journals of *Management Information Systems Quarterly* (*MISQ*), *Information Systems Research* (*ISR*), and ACM’s Transactions journals. These studies are related to the use of health care technology combined with a behavioral component that seeks to explain how AI can define patient well-being. We used the keywords “mobile health,” “mHealth,” “mobile healthcare,” “mobile sensors,” “wearable sensors,” “medical sensors,” “smartphone data,” “ML,” and “AI.”

### Step 3: Study Selection

After selecting relevant articles, we defined our study selection metrics based on the inclusion and exclusion criteria as specified in Table S1 in Multimedia Appendix 1. We eliminated several articles identified through our keyword searches that did not meet the criteria. During the last 2 years, there has been a significant increase in research in AIM [59]. In the same period, researchers have developed and used newer AI techniques, such as FL and XAI, to build predictive privacy-preserving models [20] for disease management. Therefore, we decided to limit the search for AIM articles to the last 2 years. Additionally, we considered articles where both AI and mHealth concepts were specifically used in the study design or the primary research motivation for the paper. Finally, each author independently read article summaries and abstracts to determine their eligibility for this scoping review based on the inclusion and exclusion criteria as specified in Table S1 in Multimedia Appendix 1.

### Step 4: Charting the Data

After selection of the studies for this scoping review, we segregated articles according to research streams (biomedical technology, AI, IS), type of data used (public vs proprietary), and health conditions (physical health, mental health, and general health promotion and wellness). Articles related to physical health examined the use of AIM for disease management of chronic health issues, such as asthma and diabetes, and neurological illnesses, such as Alzheimer and Parkinson disease. These severe health conditions are difficult to manage without active support from physicians, and thus, the application of mHealth sensors can be used to track patients with these conditions. Studies focused on general health promotion and wellness were related to nonchronic conditions that do not require constant medical supervision, such as leading an active lifestyle and engaging in regular exercise. mHealth sensors can notify and remind people to engage in physical activity to have an overall better level of physical health. Articles related to mental health focused on using AIM to facilitate the detection of mental health issues among the population by collecting data from personal devices.

Since public data has massive potential for enabling broader collaboration in health care technology usage and AIM adoption across organizational, national, and international boundaries, we also divided articles based on the data set they used. Studies using publicly available data sets are more effective in bringing out the potential impact of AIM and inspiring confidence among the public and medical institutions in the efficacy of AIM models.

### Step 5: Collating, Summarizing, and Reporting the Results

Finally, in the results section, we collate, summarize, and report the findings from this review. We discuss their implications for future AIM research in the discussion section and present results of the PRISMA (Preferred Reporting Items for Systematic Reviews and Meta-Analyses) technique we used to identify and select articles for this review. We also discuss the selected 37 articles using AIM capabilities for disease management and monitoring physical and mental health conditions. Furthermore, some of the articles focused on using AIM models for enhancing general health promotion and wellness of people.

## Results

### Step 1: Identifying RQs

After careful consideration and discussions, we decided to define the scope of our review paper based on the shared capabilities of AI and mHealth. Through deliberations, we decided to focus on the emerging uses of AI in the current state-of-the-art mobile health care domain. In this regard, we identified the following 3 RQs of value for both researchers and practitioners in this scoping review: (1) What are the major health conditions being researched in the AI-powered mHealth (AIM) domain? (2) How do AIM techniques use the data collected from wearable sensors and mHealth devices? (3) What are the requirements for facilitating the rapid adoption of AIM models in hospitals and medical institutions in the health care sector?

### Step 2: Identifying Relevant Studies

We initially started with 108 articles related to each of the 3 domains in this study: biomedical technology, AI, and IS. We identified 108 relevant studies in total: 101 from our selected databases (PubsOnline, n=34; e-journal archive at *MISQ*, n=27; and ACM Digital Library, n=40) and 7 articles through reference checking in search engines.

### Step 3: Study Selection

**Figure 1:**
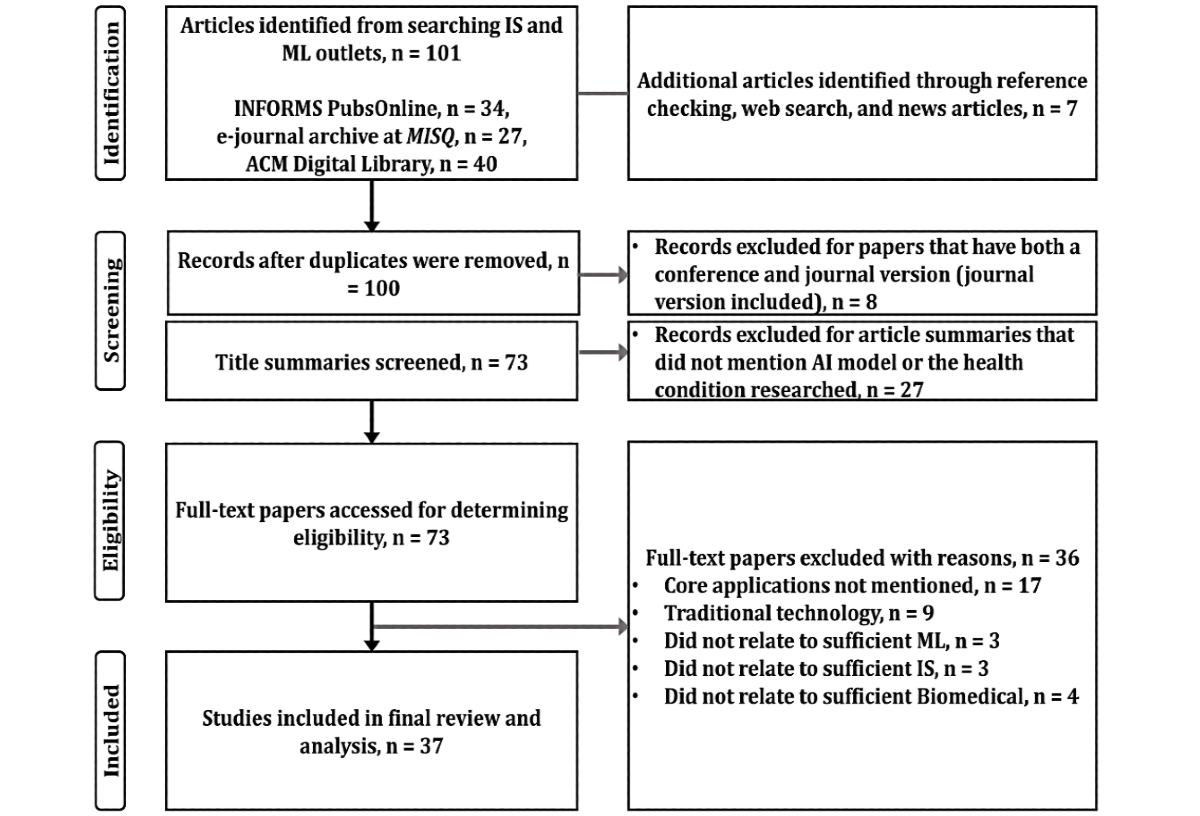
PRISMA (Preferred Reporting Items for Systematic Reviews and Meta-Analyses) flow diagram. ACM: Association for Computing Machinery; AI: artificial intelligence; IS: information systems; MISQ: Management Information Systems Quarterly; ML: machine learning.

Using the PRISMA technique depicted in Figure 1, 37 articles matched the study selection criteria for this scoping review. When selecting the articles, we proceeded to remove duplicate articles (n=8) that had both a journal and conference version (journal version included in review) and screened the title and abstract of the selected articles (n=27) to ensure sufficient AI- and mHealth-based content was present in the work. Upon final selection, we independently screened the full text of the remaining articles (n=3).

### Step 4: Charting the Data

The majority of the articles identified used mixed method research with participant-based studies that focused on using mHealth devices to collect data from people experiencing a certain health condition (asthma, diabetes, suicidal tendencies, depression, etc) and then using the data collected to train AI models to automatically detect such conditions; otherwise, they were analytical studies that applied AI models to publicly available data sets. The final set of 37 articles included in this review are presented in Table S2 in Multimedia Appendix 1.

**Figure 2:**
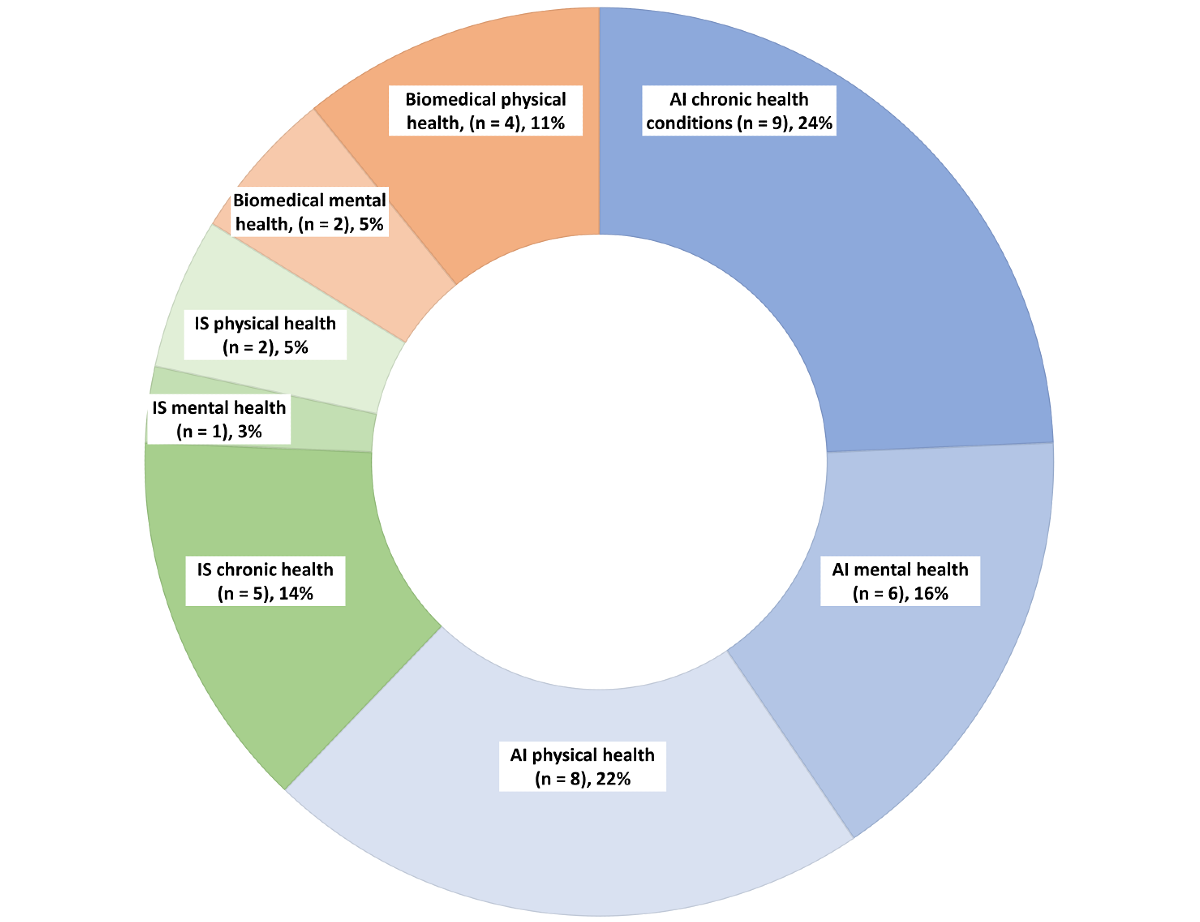
Statistics of different AI-powered mobile health domains (N=37). AI: artificial intelligence; IS: information systems.

Of the 37 articles, 31 were published in 2020 (84%), and 23 out of the 37 (62%) articles were from AI databases which represented the largest domain included in our review. Of the 23 articles within the AI domain, 17 (74%) mainly focused on physical health and chronic health conditions. Both these conditions were researched in most of the articles included in the review. Physical health articles primarily focused on using AIM models for human activity recognition and analyzing people’s activities of daily living. The data used in these studies were collected using multiple mHealth devices, such as object and motion detection sensors. However, accessing large repositories of such data is difficult because data sharing among medical institutions, hospitals, and clinical studies is often restricted [20]. This gives rise to a lack of availability of quality data sets for building AIM models, which was also observed in our review, as 28 out of the total 37 (76%) articles used proprietary data sets rather than public ones. Finally, mental health studies used a combination of qualitative techniques, such as surveys and smartphone sensors, to augment their data collection. These data were used for building predictive models that detect depression and suicidal tendencies in people. Figure 2 below presents the different metrics of the selected articles in our scoping review.

### Step 5: Collating, Summarizing, and Reporting the Results

The results from our scoping review help to answer our RQ1 about the major health conditions being researched in the AIM domain and our RQ2 about how AIM data collected from wearable sensors and mHealth devices are used by researchers. In terms of RQ1, 3 major categories are being researched in the AIM domain: mental health, physical health, and chronic health conditions. For RQ2, most of the studies use data collected from AIM devices to build and train advanced AI models that seek to detect, predict, and manage health conditions in general.

**Figure 3:**
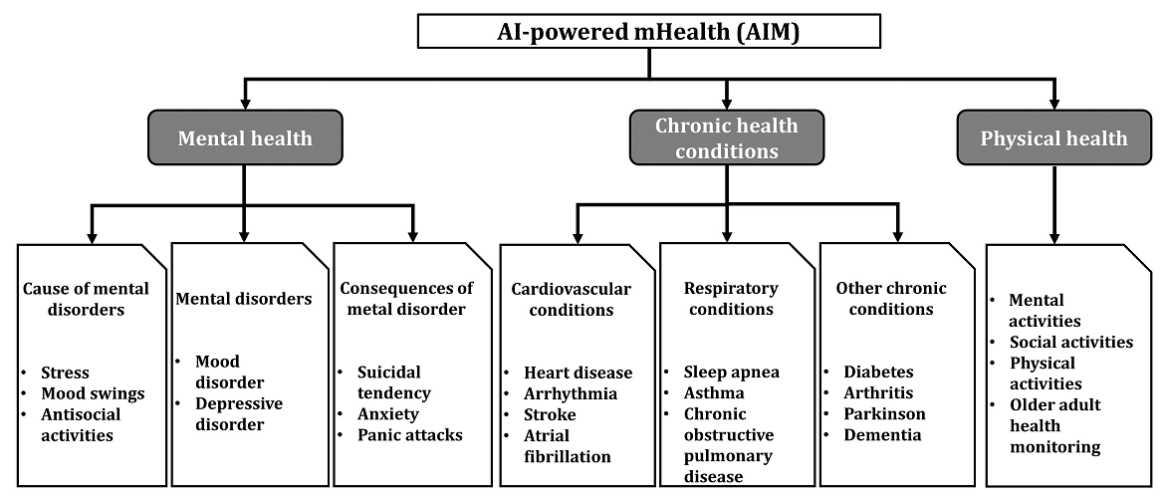
Studies in the AIM Domain. mHealth: mobile health.

As we discussed the results of our scoping review of articles on AIM, we observed the research in this domain is concentrated in 3 distinct categories of physical health, mental health, and chronic health conditions, as presented in Figure 3.

Most of the studies in the scoping review focused on chronic health conditions, such as cardiovascular conditions related to heart disease, stroke, arrhythmia, and atrial fibrillation; respiratory conditions, such as sleep apnea, asthma, and COVID-19 monitoring; and other conditions chronic conditions, such as diabetes, arthritis, and Parkinson disease. These studies explain how AIM models are used to enable greater self-management of chronic diseases by providing real-time health insights to patients and doctors [56,60]. AIM models focused on chronic health conditions are developed using heterogeneous data, including text, audio, and rhythmic body movements, collected from wearable and mobile sensors, [54,55,57,61]. Researchers note that the physiological features of people, such as their height, weight, and metabolism, can be used as data points to train personalized AIM models [62]. These models can then predict the types of chronic health conditions a person may be susceptible to (currently and in the future) [63]. For instance, researchers used AIM models to predict the likelihood of an imminent episode of Parkinson that may result in a patient falling [64]. Moreover, other researchers have demonstrated the effectiveness of AIM models in enhancing the development of preventive and precision medicine and detecting early signs of the onset of chronic conditions, such as in imminent asthma attacks [65,66].

After chronic conditions, the next major category of studies focused on mental health conditions for which research has recently increased [67]. Included were articles that sought to understand the nature, causes, and consequences of mental disorders. Studies in this category focused on using mobile sensors to predict people’s moods and behaviors while also determining the causes responsible for such a shift in them [68,69]. Research aimed at understanding the causes and consequences of mental disorders is fundamental in clinical psychiatry [70] and can be used to provide interventions to people who exhibit antisocial behavior [71]. Studies relating to the consequences of mental health disorders play a crucial role in identifying at-risk populations who might be suffering from suicidal tendencies. In some of our selected articles, researchers inferred suicidal tendencies from smartphone usage [72,73]. Smartphone usage can also help the understanding of the causes of mental health disorders. The mood of a person is indicative of the emotional state they are in. There is growing evidence to suggest people’s moods (happy, sad, etc) and their inner emotional states (anxiety, depression, panic, etc) are interlinked [74,75]. Several studies have successfully pursued this link to identify people vulnerable to suffering from mental disorders [76,77]. For instance, some authors [76] used an AIM model to find emotionally distressed people in online social networks. The model analyzes the text of users’ posts to detect the usage of negative words or phrases (eg, “I am all alone,” “I don’t want to live anymore”) that indicate if a person is feeling suicidal. Similarly, other authors [77] also used data from wearable sensors to build AIM models that detect whether people are under emotional stress and determine the underlying causes for mental disorders.

The final category of studies focused on the use of AIM devices to monitor people’s physical health. The articles studied different mental, social, and physical activities that people engage in and collected data using AIM devices. These data were used to build AIM models that detect when people were not engaging in their regular activities, such as exercising and walking. Once a lack of social or physical activity is detected, the AIM model sends out personalized suggestions encouraging people to lead an active lifestyle [78,79]. Such AIM models can also detect prolonged periods of human inactivity, which is of particular importance when monitoring the health of older adults. Studies show data related to heart rate and self-reported fatigue levels can be used to share automatic suggestions that remind people to engage in healthy exercise [80-83]. In addition, AIM devices can be used for monitoring the movements of older adults through the use of mobile sensors, such as wearable and object detection sensors [84-87]. Data collected through AIM devices can also identify human activity and encourage safe physical health practices [59,88]. For instance, during the current pandemic, researchers have used AIM devices to build models that identify and detect dangerous COVID-19 behavior, such as face touching [89,90]. Further, the use of AIM devices can help to ensure privacy and protect people’s private health data [91] by using AI techniques, such as FL, that can prevent data from being transferred outside AIM devices. These kinds of varied applications showcase the versatility of the AIM domain for ensuring the physical health of people.

## Discussion

The findings from our scoping review showcase the benefits of using AIM in applications ranging from clinical care [92] to improvements in the overall adoption and access of telehealth services [29,30]. A key finding to discuss from our work is how the recent confluence of AI and mobile wearable technology has resulted in the increase in mHealth usage [16]. Another important insight to consider is how mHealth and telehealth have emerged as reliable avenues to provide noncritical care to patients [31], which is vital during the current pandemic. This is also evident in many of the papers included in this review [78,81,83].

This review surveys the recent developments in the AIM domain, and based on our findings, we present some practical recommendations for future research. First, by using the recent advances in AI techniques of FL and XAI, AIM could facilitate an even broader expansion and provision of mHealth services which is also evidenced by its significant use during the pandemic [16]. Second, the increased adoption of such services can be helpful in building a consensus about the rules governing the usage of wearable technologies in medical institutions. Currently, hospitals use proprietary mHealth devices that do not allow data sharing even if it is for critical research purposes [20]. With the increase in adoption, different health care institutions can come together and create a shared set of rules that can enable AI-based models to study the data from across all participating institutions, thus resulting in the generation of more robust and accurate medical insights. Third, given the importance of health care access for all sections of society, governments and private institutions should promote the use of mHealth in their digital health efforts and public safety campaigns.

Given the growing importance of remote health and telehealth facilities during the COVID-19 pandemic, we conclude that it is important to facilitate greater adoption of remote health monitoring devices, protect patient privacy, and increase people’s trust in smart wearable health devices. This is because our findings show that mHealth indeed plays a significant role in shaping the future of how citizens access health care facilities in testing times such as a pandemic. In this regard, some recent AI techniques that can accelerate its adoption and enable faster implementation across hospitals, medical institutions, and users at large are discussed. The discussion of these techniques also answers RQ3 about the requirements for facilitating the rapid adoption of AIM models in hospitals and medical institutions in the health care sector and how recent AI techniques can be implemented to strengthen research in the AIM domain.

To protect patients’ privacy, ML techniques such as DL, FL, and transfer learning can effectively drive the smart health care revolution. These techniques use privacy-preserving feature engineering to translate vast amounts of biomedical data into actionable and potentially life-saving human health outcomes [93]. From the analysis of the papers included in this survey, we observe that a critical outcome of applying DL in the mHealth analytics domain is that it results in the development of powerful algorithms. These algorithms provide excellent capabilities to predict and detect diseases early, thus enabling efforts to provide preventive medicine and care to vulnerable people [94]. Since users’ data exist in isolated silos or islands across different hospitals and medical institutions, it becomes increasingly difficult for researchers working in this domain to access these data. Moreover, generalizing the performance of an ML model for a large population becomes difficult in the absence of personalized data about individuals [95]. Recent advances in FL and transfer learning show that it is a promising solution in such scenarios. It ensures data privacy, as user data never leave an institution [96]. In addition, model insights learned from one set of data can be transferred to make predictions for another set of data. When FL models are used, data remain static and situated at the source, thereby protecting privacy. The only information exchange under such models involves purely numerical representations of stochastic gradient descent. These numerical data cannot be used to reverse engineer and determine the source of data. The use of such techniques can help dismantle privacy barriers that are associated with health care data access. It can enable greater collaboration between the medical, research, and practitioner communities while ensuring faster development and integration of AIM in the health care sector. To this effect, the 3 identified research streams can act as guiding principles for providing holistic health care services that cover the mind, body, and spirit of people. It can also ensure that people receive the best possible care in the shortest time and with maximum efficacy.

The DARPA (Defense Advanced Research Projects Agency) XAI program strives to support the development of AI systems whose models can be interpreted, understood, and trusted by end users [97]. XAI is necessary for the future of AIM integration, as it can help increase the acceptability and understanding of ML techniques and models in the health care domain. With increased knowledge of AI models, we can expect an increase in the adoption rate of AIM in the health care industry, as is evidenced by various studies relating to the technology acceptance model [98]. According to this model, as the ease of use of technology increases, so does the intention and behavior of people to engage in and use the said technology. In this case, as AI models become increasingly easy to use and deploy, their widespread adoption will significantly increase hospitals’ efficacy. It will also result in better providing remote health services that depend on crucial data from patients’ wearable sensor devices. Adadi et al [99] conducted in-depth survey on XAI and note its diverse implications for the medical field in the future. They emphasize how the lack of transparency in ML models is one of the primary reasons for the nonadoption of AI in the health care industry. Peeking inside the black-box nature of AI is thus an effective way to overcome the impediments that limited knowledge and understanding place on the use of AIM. Gordon et al [100] have shown how XAI techniques can be used in surgical and operative settings in hospitals and in processing medical data for real-time clinical decision support. These models can help surgical teams to analyze, anticipate, understand, and prevent adverse intraoperative events. In another study, Payrovnaziri et al [101] surveyed how XAI specifically can be used to model real-world electronic health record data. They identify several gaps in the literature and conclude XAI has not been adequately pursued and practiced in medicine. They acknowledge there are several opportunities available where the adoption and application of XAI can significantly enhance mHealth. These have important implications for both research and practice. The recency of these surveys underscores the importance of AIM in the health care sector and provides a guideline for future research into this critical domain.

As with most scoping reviews, there are some limitations in this work. First, we only considered research from the 3 domains of biomedical technology, IS, and AI. Second, we did not consider the social aspect of AIM technology in this paper, but it is an emerging aspect of health care research. We will work to address these limitations in our future work. Third, we considered only a limited number of databases for selecting the articles and had to restrict the search so that we could focus on articles that address the latest transdisciplinary research context of AI, biomedical technology, and IS. Such work included papers that were published in niche ML and AI conference proceedings and listed within a particular database, for instance the ACM Digital Library. However, the databases we selected are comprehensive avenues for state-of-the-art research in the AIM domain and include the latest peer-reviewed research literature in the 3 streams.

Our findings from this scoping review indicate that there has recently been considerable increase in the research, practice, and adoption of mHealth and AI capabilities in the health care sector, which has resulted in significant advances in both critical and noncritical clinical care. However, certain areas still exist where there is a lack of AI research, such as in addressing mental health issues. A particular reason for this lack of research can be attributed to the nonavailability of public data sets hindering the widespread adoption of the AIM domain. A solution for this problem is to ensure collaboration and data sharing among different medical institutions. Such collaborative efforts will ensure the better utilization of AI tools by doctors, physicians, and hospitals alike. Furthermore, new and advanced AI techniques, such as FL and XAI, are rapidly being developed by researchers, and their subsequent adoption in real-world scenarios will likely have life-saving consequences in the future.

## Appendix

Multimedia Appendix 1 Selection criteria and study details of the artificial intelligence–powered mobile health (AIM) articles in the scoping review.

## Abbreviations

AAAI: Association for the Advancement of Artificial Intelligence
ACM: Association for Computing Machinery
AI: artificial intelligence
AIM: artificial intelligence–powered mobile health
DARPA: Defense Advanced Research Projects Agency
DL: deep learning
FL: federated learning
IOT: internet of things
IS: information systems
ISR: Information Systems Research
IT: information technology
mHealth: mobile health
MISQ: Management Information Systems Quarterly
ML: machine learning
NeurIPS: Neural Information Processing Systems
NIPS: Neural Information Processing Systems
PRISMA: Preferred Reporting Items for Systematic Reviews and Meta-Analyses
RQ: research question
XAI: explainable AI
