# Supplementary material for: Emerging Artificial Intelligence–Empowered mHealth: Scoping Review
Source: JMIR Mhealth Uhealth. 2022 Jun 9;10(6):e35053. doi: 10.2196/35053 (PMC9227797; doi:10.2196/35053)
Supplement: Multimedia Appendix 1 [file mhealth_v10i6e35053_app1.docx]

Appendix 1

Table 1. Selection Criteria.

| Inclusion Criteria | Exclusion Criteria |
| --- | --- |
| Documents were included if they: | Documents were excluded if they: |
| - Described advanced Machine Learning methods, techniques, or application criteria (such as Deep Learning, Collaborative Learning, Federated Learning and Reinforcement Learning) or mentioned attributes of existing ML techniques but applied them to a new research domain. | - Described traditional Machine Learning methods, techniques, or application criteria (such as CNN, Big Data Analytics and Data Mining) that were not specific in scope or related to the mHealth domain in general. |
| - Described technological setting or use in a major healthcare setting or related to chronic disease management. | - Described exploratory work without conducting experiments with data or provided general healthcare advice. |
| - Published in English language since 2019. | - Focused only on ML technologies and not on its implications for healthcare. |
| - Published with a testable or conceptual research model. | - Blogs, news articles, or far restrictive in use web content. |

Table 2. Study details of AIM articles in scoping review

| Ref | 38 |
| --- | --- |
| Stream | AI |
| Data | Proprietary |
| Health Category | Mental Health |
| Key health condition | Emergency Healthcare |
| AI Tech Used | Deep Learning |
| Purpose | Investigate the influences of technological and psychological antecedents on extrinsic and intrinsic motivations to use mobile healthcare |
| Devices | Surveys |
| Data Collected | Online health community usage data |
|  |  |
| Ref | 48 |
| Stream | AI |
| Data | Proprietary |
| Health Category | Physical Health |
| Key health condition | Human activity monitoring |
| AI Tech Used | Few-shot learning |
| Purpose | Human activity recognition. |
| Devices | Wearable sensors |
| Data Collected | Object movement data, lateral human movement data |
|  |  |
| Ref | 54 |
| Stream | AI |
| Data | Proprietary |
| Health Category | Chronic Health Conditions |
| Key health condition | Health risks caused by radiation, microgravity, |
| AI Tech Used | Clinical decision support systems (CDSS) |
| Purpose | Astronaut health monitoring |
| Devices | Wearable sensors |
| Data Collected | Heart rate |
|  |  |
| Ref | 55 |
| Stream | AI |
| Data | Proprietary |
| Health Category | Chronic Health Conditions |
| Key health condition | Cardiac arrest |
| AI Tech Used | Support vector machine (SVM) |
| Purpose | Classify agonal breathing instances in real-time within a bedroom environment. |
| Devices | Audio recording and devices |
| Data Collected | 9-1-1 cardiac arrest audio, polysomnographic sleep lab data |
|  |  |
| Ref | 56 |
| Stream | IS |
| Data | Proprietary |
| Health Category | Chronic Health Conditions |
| Key health condition | Chronic disease self monitoring. |
| AI Tech Used | Deep Learning |
| Purpose | IT Self monitoring for chronic disease management. |
| Devices | Mobile applications and insideable devices |
| Data Collected | Questionnaires and IT usage data |
|  |  |
| Ref | 57 |
| Stream | AI |
| Data | Proprietary |
| Health Category | Chronic Health Conditions |
| Key health condition | Stroke |
| AI Tech Used | Federated Learning |
| Purpose | Propose a privacy-preserving scheme to predict the risk of stroke and deploy our federated prediction model on cloud servers. |
| Devices | Electronic health records (EHRs) |
| Data Collected | EHR data integrated with mobile messaging data. |
|  |  |
| Ref | 59 |
| Stream | AI |
| Data | Proprietary |
| Health Category | Physical Health |
| Key health condition | Human activity recognition |
| AI Tech Used | Contrastive Learning |
| Purpose | Explore the adoption and adaptation of SimCLR, a contrastive learning technique for visual representations, to HAR. |
| Devices | Wearable and mobile sensors |
| Data Collected | Human activity data (lateral movements) |
|  |  |
| Ref | 60 |
| Stream | IS |
| Data | Proprietary |
| Health Category | Chronic Health Conditions |
| Key health condition | Chronic obstructive pulmonary disease (COPD) and Chronic heart failure (CHF) |
| AI Tech Used | Deep Learning |
| Purpose | Feedback for telemonitoring patients |
| Devices | Remote health systems and databases |
| Data Collected | Medical and compliance alerts |
|  |  |
| Ref | 61 |
| Stream | AI |
| Data | Proprietary |
| Health Category | Chronic Health Conditions |
| Key health condition | Cardiac conditions |
| AI Tech Used | DL and segmentation-based algorithm |
| Purpose | Detection of heart sounds to perform heart rate monitoring. |
| Devices | Audio recording and devices |
| Data Collected | Audio data related to breathing sounds |
|  |  |
| Ref | 62 |
| Stream | AI |
| Data | Public |
| Health Category | Chronic Health Conditions |
| Key health condition | Sleep Apnea |
| AI Tech Used | Deep Learning |
| Purpose | A novel late sensor fusion method is proposed which uses backward shortcut connections to improve the learning of the traditional models. |
| Devices | Sensor fusion (multiple sensors) |
| Data Collected | Heart rate, sleep patterns data |
|  |  |
| Ref | 63 |
| Stream | AI |
| Data | Proprietary |
| Health Category | Chronic Health Conditions |
| Key health condition | Diabetes |
| AI Tech Used | Personalized Learning |
| Purpose | Noninvasive Glucose Prediction |
| Devices | Wearable sensors |
| Data Collected | Glucose levels |
|  |  |
| Ref | 64 |
| Stream | AI |
| Data | Public |
| Health Category | Chronic Health Conditions |
| Key health condition | Parkinson's |
| AI Tech Used | Bi-directional recurrent neural network (RNN) |
| Purpose | Identify and rate the normality of gait patterns from streaming data and to inform clinicians of specific gait abnormalities. |
| Devices | Wearable mobile sensors |
| Data Collected | Gait Assessment video data, lateral human movement data |
|  |  |
| Ref | 65 |
| Stream | IS |
| Data | Proprietary |
| Health Category | Chronic Health Conditions |
| Key health condition | Asthma |
| AI Tech Used | Deep Learning |
| Purpose | Develop a data analytics framework for detecting abnormal inhaler use |
| Devices | Smart asthma management system (SAM) and object sensors |
| Data Collected | Bluetooth based timestamps of inhaler usage data |
|  |  |
| Ref | 66 |
| Stream | IS |
| Data | Proprietary |
| Health Category | Chronic Health Conditions |
| Key health condition | Asthma |
| AI Tech Used | Sequential Pattern mining and Random Forest, Distant Supervision |
| Purpose | Develop a data-driven framework, adapt and integrate multiple advanced machine learning techniques, and perform an empirical analysis to (1) derive characteristics of self-reported asthma patients from social media, (2) enable integration and repurposing of highly heterogeneous and commonly available datasets, and (3) uncover the sequential patterns of asthma triggers and risk factors |
| Devices | Object sensors |
| Data Collected | Social media data (text), demographic data, object detection data, inhaler usage data |
|  |  |
| Ref | 68 |
| Stream | AI |
| Data | Proprietary |
| Health Category | Mental Health |
| Key health condition | Health risks |
| AI Tech Used | Bayesian hierarchical vector autoregressive (VAR) model |
| Purpose | Predict behavioral and self-reported health outcomes on college student participants from passively collected data from their smartphones, wearable devices, and environment, as well as their self-reports. |
| Devices | Smartphones, wearable devices, |
| Data Collected | Self-reported health data, GPS location, steps walked, smartphone usage data (calls, screen time etc.) |
|  |  |
| Ref | 69 |
| Stream | AI |
| Data | Proprietary |
| Health Category | Mental Health |
| Key health condition | Cognitive Health |
| AI Tech Used | Deep Learning |
| Purpose | Develop structured models of users’ smartphone interactions to reveal differences in phone usage patterns between people with and without cognitive impairment. |
| Devices | Smartphones |
| Data Collected | Smartphone usage data (calls, notification, app logs) |
|  |  |
| Ref | 72 |
| Stream | AI |
| Data | Proprietary |
| Health Category | Mental Health |
| Key health condition | Mood swings |
| AI Tech Used | Deep Learning |
| Purpose | Study behavioral markers or daily mood using a recent dataset of mobile behaviors from high-risk adolescent populations. |
| Devices | Smartphones |
| Data Collected | Text based smartphones use data, application usage data |
|  |  |
| Ref | 73 |
| Stream | AI |
| Data | Proprietary |
| Health Category | Mental Health |
| Key health condition | Suicide Prevention |
| AI Tech Used | Deep Learning |
| Purpose | Detect behavioral shifts in psychiatric patients from unobtrusive data collected by a smartphone app. |
| Devices | Smartphone apps |
| Data Collected | Apps logs, Bluetooth, pedometer, location traces |
|  |  |
| Ref | 74 |
| Stream | Biomedical |
| Data | Public |
| Health Category | Mental Health |
| Key health condition | Depression |
| AI Tech Used | Deep Learning |
| Purpose | Combining functional brain activity and smartphone usage patterns to better understand issues related to mental health. |
| Devices | Brain imaging scans and mobile phones |
| Data Collected | Smartphone screen time data, brain imaging data |
|  |  |
| Ref | 75 |
| Stream | Biomedical |
| Data | Public |
| Health Category | Mental Health |
| Key health condition | Gneral mental health issues |
| AI Tech Used | Deep Learning |
| Purpose | Have the behaviors and mental health of the participants changed in response to the COVID-19 pandemic |
| Devices | Ecological momentary analysis |
| Data Collected | Location data, duration of phone use, number of unlocks, sedentary time. |
|  |  |
| Ref | 76 |
| Stream | IS |
| Data | Public |
| Health Category | Mental Health |
| Key health condition | Suicide Prevention |
| AI Tech Used | Rule based classification |
| Purpose | Identifies individuals who blog about their emotional distress |
| Devices | Social media posts and blogs |
| Data Collected | Text files of comments and posts on health blogs |
|  |  |
| Ref | 77 |
| Stream | AI |
| Data | Proprietary |
| Health Category | Mental Health |
| Key health condition | Affect and moods |
| AI Tech Used | Bayesian Neural Networks using Monte-Carlo (MC) Dropout |
| Purpose | To represent model uncertainties through approximations |
| Devices | Raspberry Pi |
| Data Collected | Knowledge work data, heart rate, self reported outcomes |
|  |  |
| Ref | 78 |
| Stream | AI |
| Data | Proprietary |
| Health Category | Physical Health |
| Key health condition | General health levels |
| AI Tech Used | Deep Learning |
| Purpose | Present a novel self-supervised representation learning method using activity and heart rate (HR) signals without semantic labels. |
| Devices | Wrist accelerometer & wearable ECG data |
| Data Collected | Heart rate, fitness levels data |
|  |  |
| Ref | 79 |
| Stream | AI |
| Data | Proprietary |
| Health Category | Physical Health |
| Key health condition | Activity suggestions |
| AI Tech Used | Hyperparameter Learning |
| Purpose | Propose an algorithm for providing physical activity suggestions in mHealth settings. |
| Devices | Mobile health devices |
| Data Collected | Physical activity movement data |
|  |  |
| Ref | 80 |
| Stream | AI |
| Data | Proprietary |
| Health Category | Physical Health |
| Key health condition | General health monitoring |
| AI Tech Used | Federated Learning |
| Purpose | Smart health services |
| Devices | Sensors like gyroscope, ambient light sensor, temperature, magnetic field sensor, orientation sensor, game rotation vector, linear acceleration, relative humidity, gravity, geomagnetic rotation vector |
| Data Collected | RFID, IoT communication data, push notification |
|  |  |
| Ref | 81 |
| Stream | AI |
| Data | Public |
| Health Category | Physical Health |
| Key health condition | Sedentary Lifestyle |
| AI Tech Used | Deep Learning |
| Purpose | Aimed to improve estimates of sitting time from hip-worn accelerometers used in large cohort studies by using machine learning methods developed on free-living activPAL data. |
| Devices | Hip-Worn Accelerometer |
| Data Collected | Lower body movement data to understand sitting or standing postures |
|  |  |
| Ref | 82 |
| Stream | Biomedical |
| Data | Proprietary |
| Health Category | Physical Health |
| Key health condition | Mobility |
| AI Tech Used | Web systems and Learning |
| Purpose | ROAMM framework has been developed to achieve real-time activity recognition. |
| Devices | Smart watches |
| Data Collected | Location data, physical activity data |
|  |  |
| Ref | 83 |
| Stream | Biomedical |
| Data | Proprietary |
| Health Category | Physical Health |
| Key health condition | Excess Fatigue |
| AI Tech Used | RNN |
| Purpose | Compared supervised and unsupervised machine learning approaches to gain insights on the relationship between self-reported non-pathological fatigue and multimodal sensor data. |
| Devices | Multisensory wearable device |
| Data Collected | Survey for mental fatigue, sensor data for physical activity (steps walked) |
|  |  |
| Ref | 84 |
| Stream | Biomedical |
| Data | Proprietary |
| Health Category | Physical Health |
| Key health condition | Healthy Aging |
| AI Tech Used | Deep Learning |
| Purpose | Investigate the feasibility of using IoT smart home devices in the actual residences of older adults to facilitate healthy aging |
| Devices | Multipurpose sensor (motion, temperature, luminosity, and humidity) |
| Data Collected | Motion data, ambient living conditions data. |
|  |  |
| Ref | 85 |
| Stream | Biomedical |
| Data | Proprietary |
| Health Category | Physical Health |
| Key health condition | Activity of daily living |
| AI Tech Used | Deep Learning |
| Purpose | Autonomously measure a senior's change in activity and behavior |
| Devices | Wearable devices |
| Data Collected | Hospitalization rate, fall rate, length of stay (LOS), and staff response time. |
|  |  |
| Ref | 86 |
| Stream | IS |
| Data | Proprietary |
| Health Category | Physical Health |
| Key health condition | Activity of daily living |
| AI Tech Used | Deep Learning |
| Purpose | Employ advanced deep learning algorithms to develop a novel hierarchical, multi-phase ADL recognition framework to model ADLs with different granularities. |
| Devices | Object sensors, wearable devices |
| Data Collected | Human movement data, object motion data (glass raised, glass put down, etc.) |
|  |  |
| Ref | 87 |
| Stream | IS |
| Data | Public |
| Health Category | Physical Health |
| Key health condition | Activity of daily living |
| AI Tech Used | Transfer Learning |
| Purpose | Unobtrusive smart home monitoring for senior citizens. |
| Devices | Object sensors, wearable devices |
| Data Collected | Object motion data |
|  |  |
| Ref | 88 |
| Stream | AI |
| Data | Public |
| Health Category | Chronic Health Conditions |
| Key health condition | Fitness tracking and fall detection, |
| AI Tech Used | Federated Learning |
| Purpose | Propose a framework for federated label-based aggregation, which leverages overlapping information gain across activities using Model Distillation Update. |
| Devices | Smart devices and wearable health sensors |
| Data Collected | Walking, standing sitting data points |
|  |  |
| Ref | 89 |
| Stream | AI |
| Data | Proprietary |
| Health Category | Physical Health |
| Key health condition | General health levels |
| AI Tech Used | Deep Learning |
| Purpose | Monitoring home quarantines |
| Devices | Smart Speakers |
| Data Collected | Audio data based on different voices to detect existence of multiple speakers. |
|  |  |
| Ref | 90 |
| Stream | AI |
| Data | Public |
| Health Category | Physical Health |
| Key health condition | COVID-19 |
| AI Tech Used | Deep Learning |
| Purpose | Wearable technologies and machine-learning-based algorithms can be used to automatically detect dangerous behaviors such as face touching. |
| Devices | Inertial sensors (i.e., accelerometer, magnetometer, and gyroscope) |
| Data Collected | Hand movement data (face touching) |
|  |  |
| Ref | 91 |
| Stream | AI |
| Data | Proprietary |
| Health Category | Chronic Health Conditions |
| Key health condition | General health levels |
| AI Tech Used | Federated Learning |
| Purpose | Human Mobility Prediction |
| Devices | Wearable sensors |
| Data Collected | Mobility data related to people’s movements identified through GPS and other geolocation efforts. |
|  |  |
| Ref | 102 |
| Stream | IS |
| Data | Proprietary |
| Health Category | Chronic Health Conditions |
| Key health condition | Diabetes, asthma, heart disease, |
| AI Tech Used | Multiple |
| Purpose | Chronic Diseases management |
| Devices | Systems (web and mobile), sensors devices and people centric wearables. |
| Data Collected | Blood pressure, heart rate, glucose levels etc. |
